# Memory B cell responses to Omicron subvariants after SARS-CoV-2 mRNA breakthrough infection in humans

**DOI:** 10.1084/jem.20221006

**Published:** 2022-09-23

**Authors:** Zijun Wang, Pengcheng Zhou, Frauke Muecksch, Alice Cho, Tarek Ben Tanfous, Marie Canis, Leander Witte, Brianna Johnson, Raphael Raspe, Fabian Schmidt, Eva Bednarski, Justin Da Silva, Victor Ramos, Shuai Zong, Martina Turroja, Katrina G. Millard, Kai-Hui Yao, Irina Shimeliovich, Juan Dizon, Anna Kaczynska, Mila Jankovic, Anna Gazumyan, Thiago Y. Oliveira, Marina Caskey, Christian Gaebler, Paul D. Bieniasz, Theodora Hatziioannou, Michel C. Nussenzweig

**Affiliations:** 1 Laboratory of Molecular Immunology, The Rockefeller University, New York, NY; 2 Laboratory of Retrovirology, The Rockefeller University, New York, NY; 3 Howard Hughes Medical Institute, Chevy Chase, MD

## Abstract

Individuals who receive a third mRNA vaccine dose show enhanced protection against severe COVID-19, but little is known about the impact of breakthrough infections on memory responses. Here, we examine the memory antibodies that develop after a third or fourth antigenic exposure by Delta or Omicron BA.1 infection, respectively. A third exposure to antigen by Delta breakthrough increases the number of memory B cells that produce antibodies with comparable potency and breadth to a third mRNA vaccine dose. A fourth antigenic exposure with Omicron BA.1 infection increased variant-specific plasma antibody and memory B cell responses. However, the fourth exposure did not increase the overall frequency of memory B cells or their general potency or breadth compared to a third mRNA vaccine dose. In conclusion, a third antigenic exposure by Delta infection elicits strain-specific memory responses and increases in the overall potency and breadth of the memory B cells. In contrast, the effects of a fourth antigenic exposure with Omicron BA.1 are limited to increased strain-specific memory with little effect on the potency or breadth of memory B cell antibodies. The results suggest that the effect of strain-specific boosting on memory B cell compartment may be limited.

## Teaser

Wang et al. analyze memory B cell and antibody responses in SARS-CoV-2 mRNA vaccines to breakthrough infections with Delta or Omicron BA.1 variants. Breakthrough infection after two or three doses of mRNA vaccination was comparable to three doses of vaccination in eliciting broad and potent memory B cells. The findings provide insights on broad and strain-specific memory responses after mRNA vaccination with Wuhan-Hu-1.

## Introduction

Severe acute respiratory syndrome coronavirus (SARS-CoV-2) emerged in late 2019, causing a global pandemic with >500 million infections and >6 million deaths reported to date. Over the course of the pandemic, SARS-COV-2 has continued to evolve, resulting in substantial genetic distance between circulating variants and the initial viral sequence on which vaccines are based. Several of these circulating variants have been designated variants of concern (VoC) and have led to successive waves of infection, most notably by VoCs Alpha (Supasa et al., 2021), Delta (Liu et al., 2021), and Omicron (Dejnirattisai et al., 2022).

Higher rates of re-infection and vaccine-breakthrough infection with the Delta and Omicron variants highlighted the potential for immune escape from neutralizing antibody responses resulting in reduced vaccine efficacy against SARS-CoV-2 infection (Cao et al., 2022; Cele et al., 2022; Dejnirattisai et al., 2022; Gaebler et al., 2022; Hachmann et al., 2022; Kuhlmann et al., 2022; Liu et al., 2021). With the emergence of Omicron BA.1 and related lineages, infection has surged worldwide, and these new variants account for over 95% of recent COVID-19 cases. To date, BA.2.12.1 variant (a BA.2 lineage) contributes 59% of new cases in the United States, while BA.4 and BA.5 caused a fifth wave of COVID-19 infection in South Africa. Nevertheless, vaccine-elicited immunity continues to provide robust protection against severe disease, even in the face of viral variants (Andrews et al., 2022; Madhi et al., 2022; Wolter et al., 2022; World Health Organization, 2022).

Previous studies have shown that Delta or Omicron breakthrough infection boosts plasma neutralizing activity against both the Wuhan-Hu-1 strain and the infecting variant, which might suggest recall responses of cross-reactive vaccine-induced memory B cells (MBCs; Kaku et al., 2022; Quandt et al., 2022; Richardson et al., 2022; Seaman et al., 2022; Servellita et al., 2022). However, far less is known about the memory antibody responses after breakthrough infection. Here, we report on the development of antibodies produced by MBCs in a cohort of vaccinated individuals who were subsequently infected with Delta or Omicron.

## Results

**Figure 1.**
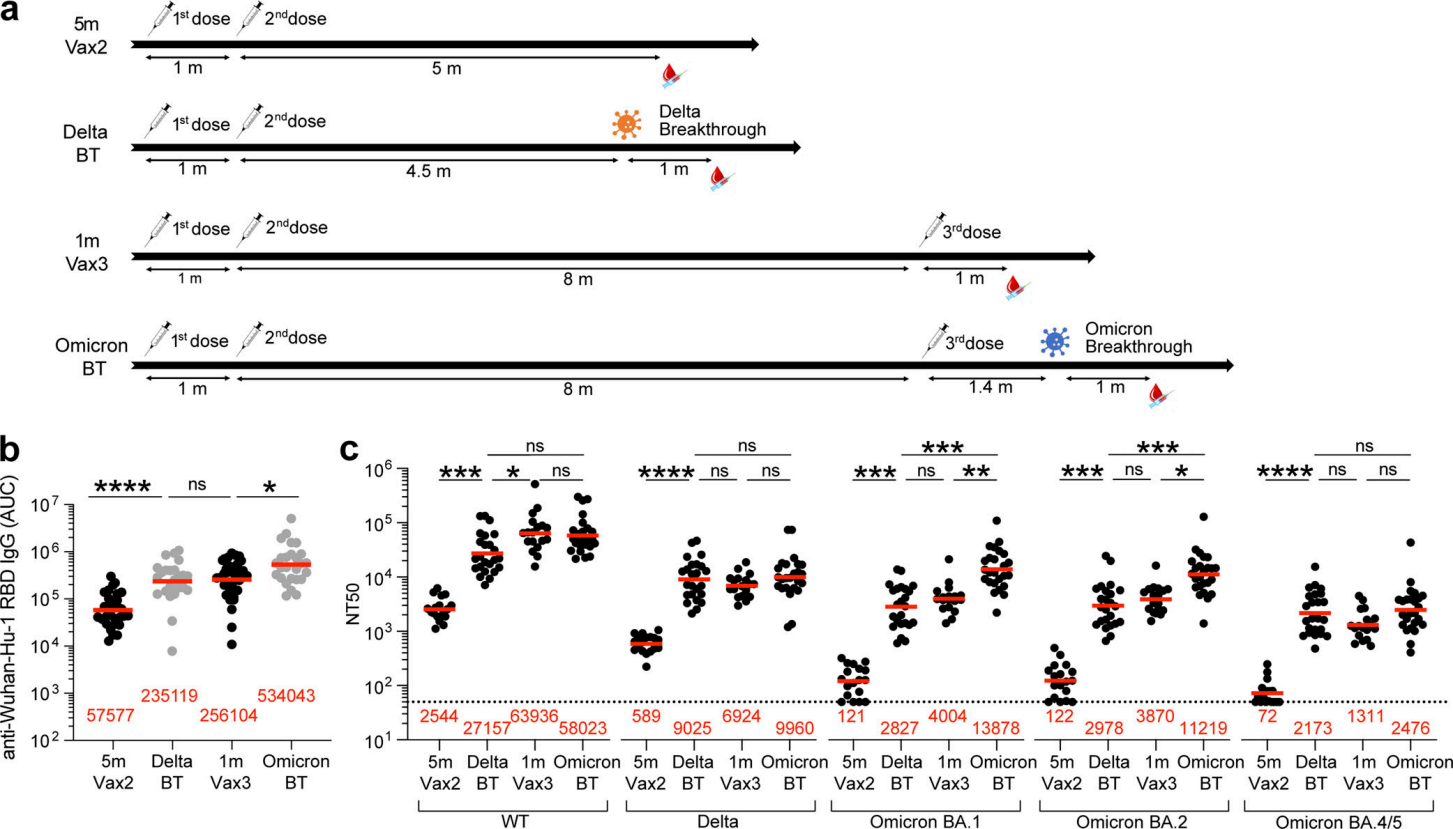
**Plasma ELISAs and neutralizing activity. (a)** Diagram shows blood donation schedules for vaccinated-only individuals 5 mo (m) after the second dose (Vax2, top; Cho et al., 2021), Delta breakthrough infection after Vax2 (Delta BT, second from top), and vaccinated-only individuals 1 mo after the third dose (Vax3, second from bottom; Muecksch et al., 2022) and Omicron breakthrough infection after Vax3 (Omicron BT, bottom). **(b)** Graph shows area under the curve (AUC) for plasma IgG antibody binding to WT SARS-CoV-2 RBD after Vax2 (Cho et al., 2021), Delta BT for *n* = 24 samples, Vax3 (Muecksch et al., 2022) and Omicron BA.1 BT for *n* = 26 samples. **(c)** Plasma neutralizing activity against indicated SARS-CoV-2 variants after Vax2 (Cho et al., 2021) for *n* = 18 samples, Delta BT for *n* = 24 samples, Vax3 (Muecksch et al., 2022) for *n* = 18 samples and Omicron BA.1 BT for *n* = 26 samples. WT and Omicron BA.1 NT_50_ values are derived from two previous reports (Gaebler et al., 2022; Schmidt et al., 2022). See Materials and methods for a list of all substitutions/deletions/insertions in the spike variants. All experiments were performed at least in duplicate. Red bars and values in panels a–c represent geometric mean values. Statistical significance in panels b and c was determined by two-tailed Kruskal–Wallis test with subsequent Dunn’s multiple comparisons. *, P ≤ 0.05; **, P ≤ 0.01; ***, P ≤ 0.001; ****, P ≤ 0.0001; ns, not significant.

Between August 13, 2021, and February 3, 2022, we recruited individuals who had been vaccinated with two or three doses of an mRNA vaccine and experienced breakthrough infections with Delta (*n* = 24, age range 21–63 yr, median age = 30 yr; 67% male, 33% female) or Omicron (*n* = 29, age range 22–79 yr, median age = 33.5 yr; 53% male, 47% female; Table S1; Gaebler et al., 2022). Volunteers received either the Moderna (mRNA-1273; *n* = 12), Pfizer-BioNTech (BNT162b2; *n* = 33), or combination (Moderna-Pfizer; *n* = 8) mRNA vaccine (Table S1). Samples from Delta and Omicron BA.1 breakthrough participants were collected a median 26.5 d (range 0–60) or 24 d (range 10–37) after positive test for infection, respectively (Table S1). As a result, Delta breakthrough samples were collected at a median of 5.5 mo (range 109–211 d) after second vaccination, and Omicron BA.1 samples were collected at median of 2.4 mo (range 26–141 d) after third vaccination (Fig. 1 a; see Materials and methods and Table S1). For two participants, paired samples were collected shortly after their third vaccine dose and again after Omicron BA.1 breakthrough infection (Table S1).

### Plasma binding and neutralization

**Figure S1.**
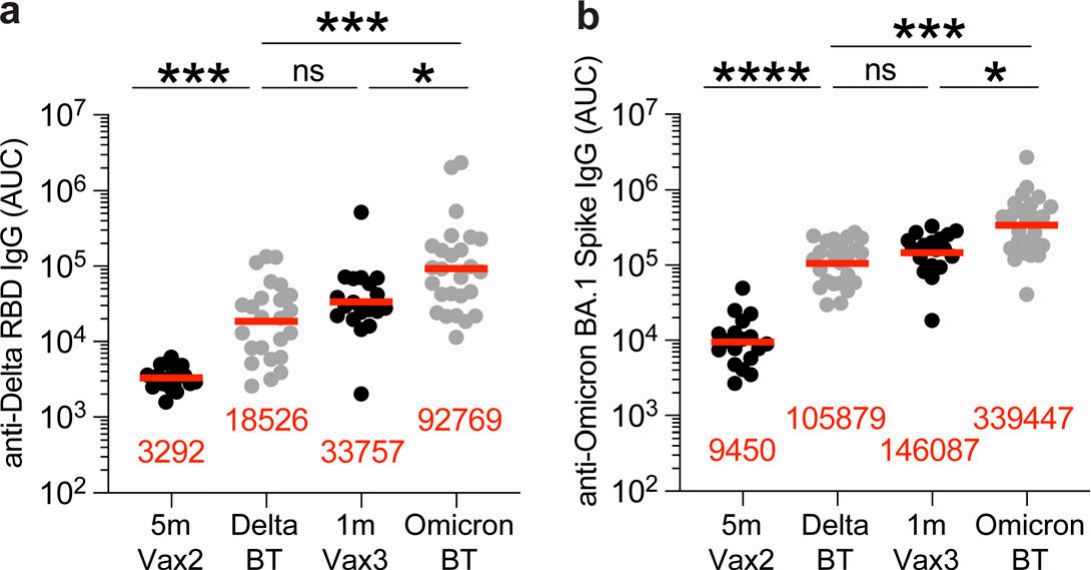
**Plasma ELISA. (a and b)** Graph shows area under the curve (AUC) for plasma IgG binding to (a) SARS-CoV-2 Delta-RBD and (b) Omicron-Spike for vaccinated individuals after Vax2 (Cho et al., 2021), Delta breakthrough (Delta BT, *n* = 24), and vaccinated individuals after Vax3 (Muecksch et al., 2022) and Omicron breakthrough infection after Vax3 (Omicron BT, *n* = 26). All experiments were performed at least in duplicate and repeated twice. Red bars and values represent geometric mean values. Statistical significance in panels a and b was determined by two-tailed Kruskal–Wallis test with subsequent Dunn’s multiple comparisons. *, P ≤ 0.05; ***, P ≤ 0.001; ****, P ≤ 0.0001; ns, not significant.

Plasma IgG antibody titers against SARS-CoV-2 Wuhan-Hu-1-(WT), or Delta-receptor binding domain (RBD), and Omicron BA.1-Spike were measured by ELISA (Wang et al., 2021c). Anti–WT-RBD IgG titers were significantly increased after Delta breakthrough infection in individuals who received two doses of mRNA vaccine (Delta BT), compared to vaccinated individuals who did not experience infection (5 m-Vax2; P < 0.0001, Vax2 [Cho et al., 2021] vs. Delta; Fig. 1 b and Table S1). Similarly, there was a twofold increase in geometric mean IgG-binding titers against WT-RBD after Omicron BA.1 breakthrough infection (Omicron BT) in individuals who received three doses of mRNA vaccine, compared to vaccinated individuals who were not infected after the third vaccine dose (1 m-Vax3; P = 0.033, Vax3 [Muecksch et al., 2022] vs. Omicron; Fig. 1 b and Table S1). Individuals who experienced Omicron BA.1 infection exhibited higher anti–Delta-RBD and anti–Omicron BA.1-Spike IgG binding titers than individuals with Delta breakthrough infection or those receiving three mRNA vaccine doses (Fig. S1; anti-Delta RBD: P < 0.0001, Delta BT vs. Omicron BT, P = 0.047, Vax3 vs. Omicron BT; anti-Omicron BA.1 Spike: P < 0.0001, Delta BT vs. Omicron BT, P = 0.021, Vax3 vs. Omicron BT).

Plasma-neutralizing activity in 49 participants was measured using HIV-1 pseudotyped with the WT SARS-CoV-2 spike protein (Cho et al., 2021; Wang et al., 2021c; Fig. 1 c and Table S1). Compared to individuals who received two mRNA vaccine doses (Cho et al., 2021), Delta breakthrough infection resulted in 11-fold increased geometric mean half-maximal neutralizing titer (NT_50_; P = 0.0003, Vax2 vs. Delta; Fig. 1 c). However, the resulting geometric mean NT_50_ was lower than after the third mRNA vaccine dose (P = 0.03, Delta vs. Vax3; Fig. 1 c). Notably, the NT_50_ against WT after Omicron breakthrough was not significantly different from individuals who received a third vaccine dose (Muecksch et al., 2022; P > 0.99, Vax3 vs. Omicron; Fig. 1 c).

Plasma-neutralizing activity was also assessed against SARS-CoV-2 Delta, Omicron BA.1, BA.2, and BA.4/5 variants using viruses pseudotyped with appropriate variant spike proteins.

Delta breakthrough infection resulted in 15-fold increased neutralizing titers against Delta compared to two-dose vaccinated-only individuals (P < 0.0001; Fig. 1 c) with resulting titers being comparable to three-dose vaccinated individuals before and after Omicron breakthrough infection (P > 0.99; Fig. 1 c). While Delta breakthrough infection also increased neutralizing titers against Omicron BA.1, BA.2, and BA4/5 (P = 0.0003, P = 0.002, and P < 0.0001, respectively; Fig. 1 c), the titers were not significantly different from titers observed in three-dose vaccinated individuals (Muecksch et al., 2022; P > 0.99, P > 0.99, and P = 0.61, respectively; Fig. 1 c). Conversely, Omicron breakthrough infection after three-dose vaccination resulted in a further 3.5-fold and 2.9-fold increase of Omicron BA.1 and BA.2 neutralizing titers, respectively, when compared to three-dose vaccinated-only individuals (Muecksch et al., 2022; P = 0.005 and P = 0.019, respectively; Fig. 1 c). Omicron BA.4/5 showed the highest neutralization resistance of all variants tested, resulting in low geometric mean neutralizing titers in plasma samples obtained after the second vaccine dose (NT_50_ = 72; Fig. 1 c). Nevertheless, individuals who had at least three antigen exposures (Delta breakthrough, Vax3, and Omicron breakthrough) were able to neutralize Omicron BA.4/5 with NT_50_s of 2,173, 1,311, and 2,476, respectively, at the time points assayed.

### MBCs

**Figure 2.**
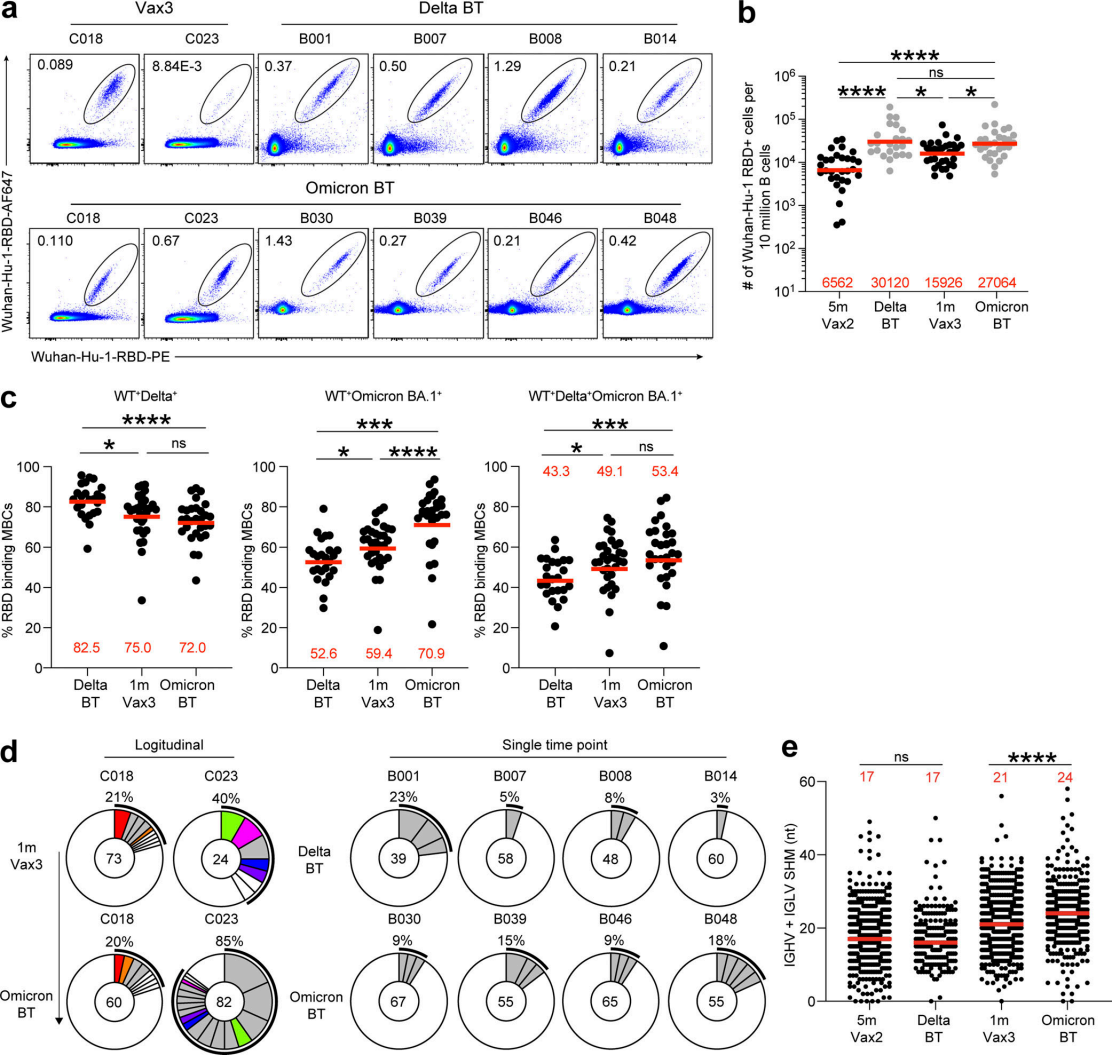
**Anti–SARS-CoV-2 RBD MBCs after breakthrough infection. (a)** Representative flow cytometry plots indicating PE-WT-RBD and AlexaFluor-647–WT-RBD binding MBCs from four individuals after Delta breakthrough infection following Vax2 (Delta BT), two individuals 1 mo after Vax3, and six individuals after Omicron BA.1 breakthrough infection following Vax3 (Omicron BT). **(b)** The number of WT RBD–specific B cells is indicated, 5 mo (m) after Vax2 (Cho et al., 2021), Delta BT (*n* = 24), 1 mo after Vax3 (Muecksch et al., 2022), and Omicron BT (*n* = 29). **(c)** Graphs showing the percentage of WT-, Delta-, and Omicron BA.1-RBD cross-binding B cells determined by flow cytometer in vaccinees (Vax3) and breakthrough individuals (Delta BT or Omicron BT; see also Fig. S2 b). **(d)** Pie charts show the distribution of IgG antibody sequences obtained from WT-specific MBCs from: two individuals assayed sequentially 1 mo after the third mRNA dose (Vax3) and followed by an Omicron infection (Omicron-BT; left); four individuals after Delta breakthrough (Delta); and four individuals after Omicron breakthrough (Omicron). The number inside the circle indicates the number of sequences analyzed for the individual denoted above the circle. Pie slice size is proportional to the number of clonally related sequences. The black outline and associated numbers indicate the percentage of clonal sequences detected at each time point. Colored slices indicate persisting clones (same IGHV and IGLV genes, with highly similar CDR3s) found at more than one time point within the same individual. Gray slices indicate clones unique to the time point. White slices indicate sequences isolated only once per time point. **(e)** Number of nucleotide somatic hypermutations (SHM) in IGHV + IGLV in WT-RBD–specific sequences after Delta or Omicron breakthrough infection, compared to 5 mo after Vax2, and 1 mo after Vax3. All experiments were performed at least in duplicate and repeated twice. Red bars and numbers in panels b and c represent geometric mean, and in panel e represent median values. Statistic analysis in panels b and c was determined by two-tailed Kruskal–Wallis test with subsequent Dunn’s multiple-comparisons test and in panel e by two-tailed Mann–Whitney test. *, P ≤ 0.05; ***, P ≤ 0.001; ****, P ≤ 0.0001; ns, not significant.

**Figure S2.**
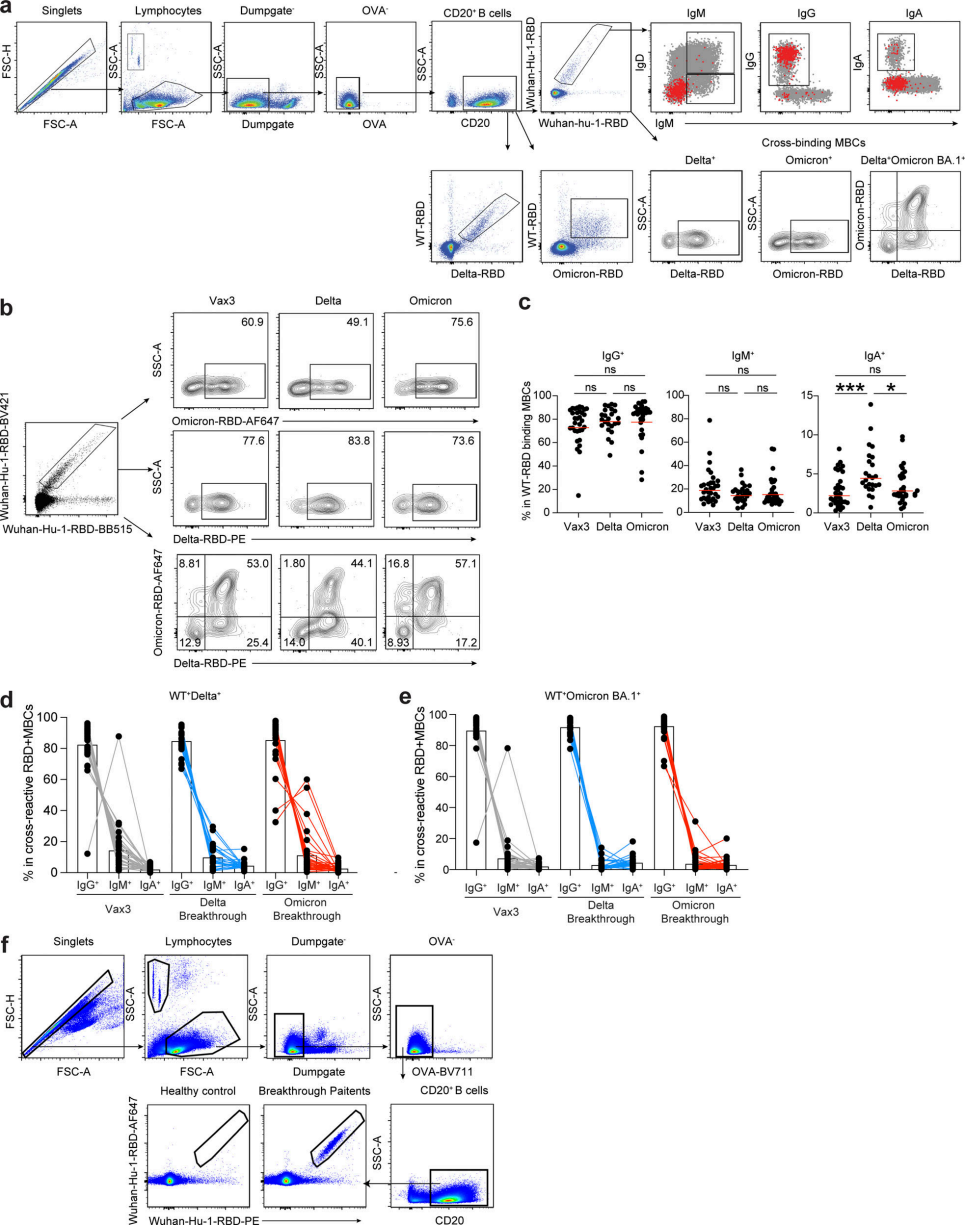
**Flow cytometry. (a and b)** Gating strategy for phenotyping. Gating was on lymphocytes singlets that were CD20^+^ and CD3^−^CD8^−^CD16^−^Ova^−^. Anti-IgG, IgM, and IgA antibodies were used for B cell phenotype analysis. Antigen-specific cells were detected based on binding to WT RBD-PE^+^ and RBD-AF647^+^, or to Delta-RBD and Omicron BA.1-RBD. **(c–e)** Graphs show the frequency of IgM, IgG, and IgA isotypes expression in (c) WT RBD^+^ MBCs, (d) WT^+^Delta^+^ RBD-binding MBCs, and (e) WT^+^Omicron BA.1^+^ RBD-binding MBCs cells. **(f)** Gating strategy for single-cell sorting for CD20^+^ B cells for WT RBD-PE and RBD-AF647. All experiments were performed at least in duplicate and repeated twice. Statistical significance in panel c was determined by two-tailed Kruskal–Wallis test with subsequent Dunn’s multiple comparisons. *, P ≤ 0.05; ***, P ≤ 0.001; ns, not significant.

mRNA vaccines elicit MBCs that can contribute to durable immune protection from serious disease by mediating rapid and anamnestic antibody response (Muecksch et al., 2022; Victora and Nussenzweig, 2022). To better understand the MBC compartment after Delta or Omicron BA.1 breakthrough infection in vaccinated individuals, we enumerated RBD-specific MBCs using Alexa Fluor 647 (AF647)– and phycoerythrin (PE)-labeled WT RBD of the SARS-CoV-2 spike protein by flow cytometry (Fig. 2 a and Fig. S2). The number of WT RBD-specific MBCs after Delta breakthrough infection was significantly higher than after the second or third vaccine dose (Delta vs. Vax2, P < 0.0001, and Delta vs. Vax3, P = 0.011; Fig. 2 b). Omicron BA.1 breakthrough infection elicited a 1.7-fold increase in the number of MBCs compared to individuals who received three vaccine doses (Vax3 vs. Omicron, P = 0.013; Fig. 2 b). Consistent with previous reports (Goel et al., 2022; Kaku et al., 2022; Nutalai et al., 2022; Park et al., 2022), flow cytometry showed that a larger fraction of the MBCs developing after the third vaccine dose or Omicron BA.1 breakthrough infection were cross-reactive with WT-, Delta-, and Omicron BA.1-RBDs than after Delta breakthrough infection (Fig. 2 c). Additional phenotyping indicated that RBD-specific MBCs elicited by Vax3 or Delta or Omicron BA.1 breakthrough infection showed higher frequencies of IgG than IgM and IgA expressions (Fig. S2, c–e).

To examine the specificity and neutralizing activity of the antibodies produced by MBCs, we purified and sequenced antibody genes in individual WT-RBD–specific B cells from 10 individuals who experienced Delta or Omicron BA.1 breakthrough infection, following the second or third vaccine dose, respectively (Fig. 2 d, Fig. S2 f, and Table S1), including two participants for whom paired samples were collected shortly after their third vaccine dose and after subsequent Omicron BA.1 breakthrough infection.

**Figure S3.**
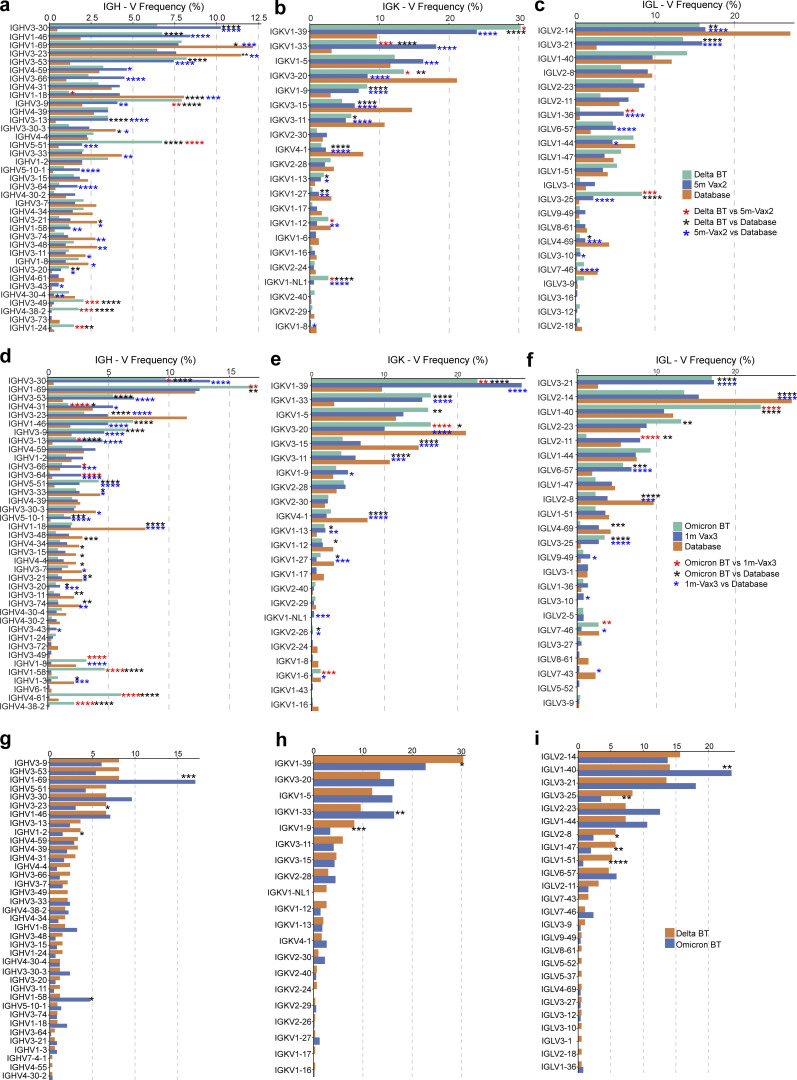
**Frequency distribution of human V genes.** **(a–c)** Comparison of the frequency distribution of human V genes for heavy chain and light chains of anti-RBD antibodies from this study and from a database of shared clonotypes of human B cell receptor generated by Soto et al. (2019). Graph shows relative abundance of human *IGHV* (left panel), *IGKV* (middle panel), and *IGLV* (right panel) genes in Sequence Read Archive accession SRP010970 (orange), antibodies obtained from Delta breakthrough infection (green), and Vax2 (blue). **(d–f)** Same as panels a–c. Graph shows relative abundance of human *IGHV* (left panel), *IGKV* (middle panel), and *IGLV* (right panel) genes in Sequence Read Archive accession SRP010970 (orange), antibodies obtained from Omicron BA.1 breakthrough infection (green), and Vax3 (blue). **(g–i)** Graph shows relative abundance of human *IGHV* (left panel), *IGKV* (middle panel), and *IGLV* (right panel) genes of antibodies obtained from Delta breakthrough infection (orange) and from Omicron BA.1 breakthrough infection (blue). Statistical significance was determined by two-sided binomial test. *, P ≤ 0.05; **, P ≤ 0.01; ***, P ≤ 0.001; ****, P ≤ 0.0001.

**Figure S4.**
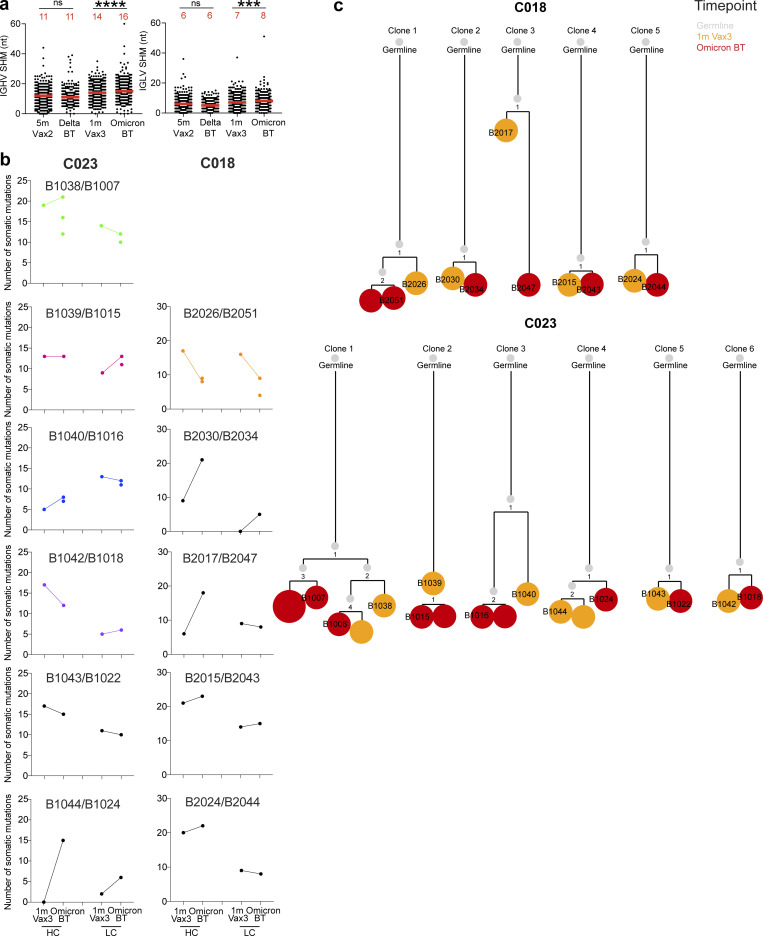
**Antibody gene somatic hypermutations analysis and phylogenetic trees. (a)** Number of nucleotide somatic hypermutations (SHM) in *IGHV* and *IGLV* in WT-RBD–specific sequences, separately after Delta or Omicron breakthrough infection, to Vax2 (Cho et al., 2021) and Vax3 (Muecksch et al., 2022). Red bars and numbers in panel a represent median value. **(b)** The number of somatic nucleotide mutations found in clonally related families found in 1 mo after Vax3 and following Omicron breakthrough infection from patients C018 and C023. Color of dot plots match the color of pie slices within the donut plot (Fig. 2 d), which indicate persisting clones. **(c)** The phylogenetic tree graph shows clones from C018 and C023, representing the clonal evolution of RBD-binding MBCs and derived antibodies obtained from the third mRNA vaccine and the following Omicron BA.1 breakthrough infection. ***, P ≤ 0.001; ****, P ≤ 0.0001; ns, not significant.

686 paired heavy- and light-chain anti-RBD antibody sequences were obtained (Fig. 2 d and Table S2). Clonally expanded WT-RBD–specific B cells represented 9% of all MBCs after Delta breakthrough infection and 28% of the repertoire after Omicron BA.1 breakthrough infection (Fig. 2 d and Table S2). Similar to mRNA vaccinees (Cho et al., 2021; Muecksch et al., 2022; Wang et al., 2021c), several sets of VH genes including *VH3-30* and *VH3-53* were over-represented in Delta or Omicron BA.1 breakthrough infection (Fig. S3). In addition, *VH3-49*, *VH4-38*, and *VH1-24* were exclusively over-represented after Delta breakthrough infection (Fig. S3 a), while *VH1-69*, *VH1-58*, *VH4-61*, and *VH4-38* were specifically over-represented after Omicron BA.1 breakthrough infection (Fig. S3 d). These results suggest that Delta and Omicron BA.1 breakthrough infections elicit variant-specific memory antibody responses. While levels of somatic mutation in MBCs emerging after Delta breakthrough infection were comparable to those after the second vaccine dose, significantly higher numbers of somatic mutations were noted following Omicron BA.1 breakthrough infection compared to the third vaccine dose (P < 0.0001; Fig. 2 e and Fig. S4, a and b). Moreover, phylogenetic analysis revealed that sequences found after the third vaccine dose and following Omicron BA.1 breakthrough infection were intermingled and similarly distant from their unmutated common ancestors (Fig. S4 c).

### mAbs

**Figure 3.**
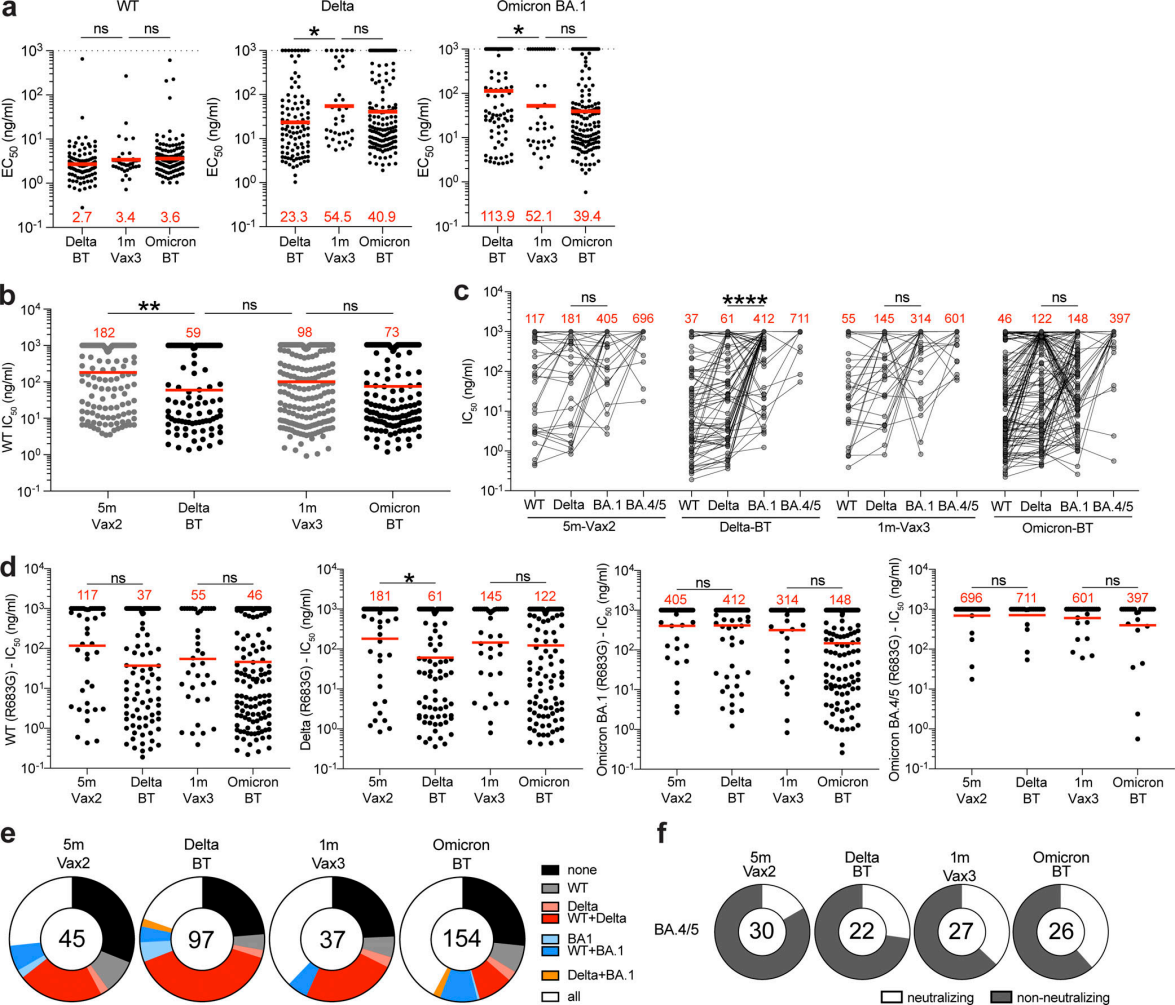
**Anti–SARS-CoV-2 RBD mAbs. (a)** Graphs show EC_50_ of *n* = 342 mAbs measured by ELISA against WT-RBD, Delta-RBD, and Omicron BA.1-spike protein. Antibodies were obtained from MBCs after Delta breakthrough (Delta BT), after mRNA Vax3, and Omicron breakthrough (Omicron BT). **(b)** Graph shows anti–SARS-CoV-2–neutralizing activity of mAbs measured by a SARS-CoV-2 pseudotype virus neutralization assay using WT SARS-CoV-2 pseudovirus. IC_50_ values for all antibodies including the 288 reported and tested herein, and 350 previously reported (Cho et al., 2021; Muecksch et al., 2022). **(c and d)** Graphs show IC_50_s of mAbs against WT, Delta-RBD, and Omicron BA.1 SARS-CoV-2 pseudoviruses. Each dot represents one antibody, where 333 total antibodies were tested including the 288 reported herein, and 45 5 m-Vax2 antibodies previously reported (Cho et al., 2021; Muecksch et al., 2022). Red values represent geometric mean values. In addition, 105 antibodies distributed over all four cohorts were also tested against Omicron BA.4/5 psuedovirus. **(e)** Ring plots show fraction of neutralizing (IC_50_ <1,000 ng/ml) antibodies against WT, Delta-RBD, and Omicron BA.1 SARS-CoV-2 pseudoviruses, and non-neutralizing (IC_50_ >1,000 ng/ml) antibodies from each time point. **(f)** Ring plots show fraction of mAbs that are neutralizing (IC_50_ 1–1,000 ng/ml, white) or non-neutralizing (IC_50_ >1,000 ng/ml, black) against Omicron BA.4/5. Number in inner circles indicates number of antibodies tested. The deletions/substitutions corresponding to viral variants used in panels c–f were incorporated into a spike protein that also includes the R683G substitution, which disrupts the furin cleavage site and increases particle infectivity. Neutralizing activity against mutant pseudoviruses was compared to a WT SARS-CoV-2 spike sequence (NC_045512), carrying R683G where appropriate. All experiments were performed at least in duplicate and repeated twice. Red bars and values in panels a, b, and d represent geometric mean values. Statistical significance in panels a and b was determined by two-tailed Kruskal–Wallis test with subsequent Dunn’s multiple comparisons, in panel c was determined by two-tailed Wilcoxon test, and in panel d was determined by two-tailed Mann–Whitney test. *, P ≤ 0.05; **, P ≤ 0.01; ****, P ≤ 0.0001; ns, not significant.

**Figure S5.**
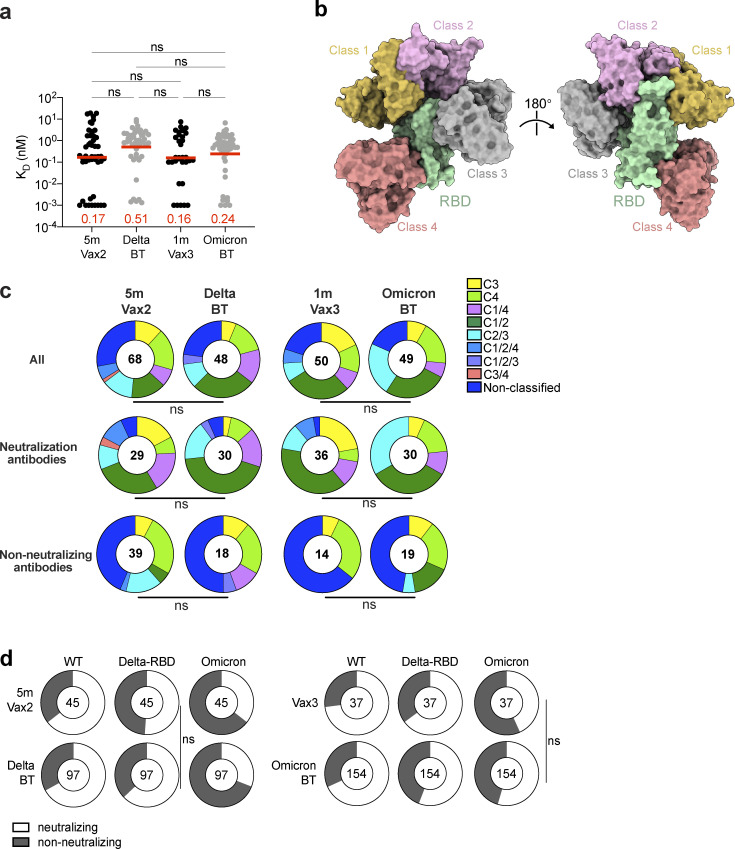
**mAb affinity, epitopes, and neutralizing breadth. (a)** Graph showing affinity measurements (K_D_s) for WT RBD measured by BLI for antibodies cloned from vaccinated individuals after Delta or Omicron breakthrough infection, compared to Vax2 (Cho et al., 2021) and Vax3 (Muecksch et al., 2022). **(b)** Diagram represents binding poses of antibodies used in BLI competition experiments on the RBD epitope. **(c)** Results of epitope mapping performed by competition BLI, comparing mAbs cloned from vaccinated individuals after Delta (*n* = 48) or Omicron BA.1 (*n* = 49) breakthrough infection, compared to Vax2 (Cho et al., 2021) and Vax3 (Muecksch et al., 2022). Pie charts show the distribution of the antibody classes among all RBD-binding antibodies (upper panel), WT neutralizing antibodies only (middle panel), or non-neutralizing antibodies only (lower panel). Red bars represent geometric mean values. Statistical significance was determined using (a) two-tailed Kruskal–Wallis test with subsequent Dunn’s multiple comparisons; or (c) two-tailed Chi-square test. **(d)** Ring plots show fraction of mAbs in Fig. 3, c–e that are neutralizing (IC_50_ 1–1,000 ng/ml, white) or non-neutralizing (IC_50_ > 1,000 ng/ml, black) for mutant or variant SARS-CoV-2 pseudovirus indicated across the top at the time point indicated to the left. The number inside of the circle indicates the number of antibodies tested. The deletions/substitutions corresponding to viral variants were incorporated into a spike protein that also includes the R683G substitution, which disrupts the furin cleavage site and increases particle infectivity. Neutralizing activity against mutant pseudoviruses was compared to a WT SARS-CoV-2 spike sequence (NC_045512), carrying R683G where appropriate. All experiments were performed at least in duplicate and repeated twice. Statistical significance in panel d was determined by using two-sided Fisher’s exact test. ns, not significant.

338 anti-RBD mAbs were expressed and tested for binding by ELISA, including 115 antibodies obtained after Delta breakthrough infection (Delta BT), 40 isolated from two longitudinal samples after their third vaccine dose in individuals who were subsequently infected (Vax3), and 183 antibodies obtained from six individuals after Omicron BA.1 breakthrough infection (Omicron). 85% (*n* = 288) of the antibodies bound to the WT RBD with a half-maximal concentration (EC_50_) of <1,000 ng/ml (Table S3). The geometric mean ELISA EC_50_ against WT RBD for the mAbs obtained from Vax3 was comparable to those found after Delta or Omicron BA.1 breakthrough infections (Fig. 3 a). In addition, antibodies isolated after both Delta and Omicron breakthrough infection showed comparable affinity for WT RBD to antibodies obtained from Vax3 when measured by biolayer interferometry (BLI; Fig. S5 a). However, when tested against Delta-RBD antibodies obtained after Delta breakthrough infection showed increased binding compared to those after Vax3. In contrast, there was no statistically significant difference in binding to Omicron BA.1-Spike by Omicron and Vax3 antibodies (Fig. 3 a).

Anti-RBD antibodies elicited by mRNA vaccination target four structurally defined classes of epitopes on the SARS-CoV-2 RBD (Barnes et al., 2020a; Cho et al., 2021; Muecksch et al., 2022; Yuan et al., 2020). To compare the epitopes recognized by anti-RBD memory antibodies elicited by mRNA vaccination (Muecksch et al., 2022) and breakthrough infection, we performed BLI competition experiments. A preformed antibody–RBD complex was exposed to a second antibody recognizing one of the four classes of structurally defined antigenic sites (C105 as Class 1; C144 as Class 2; C135 as Class 3; and C2172 as Class 4; Barnes et al., 2020a; Muecksch et al., 2022; Fig. S5b). Antibodies obtained after Delta (*n* = 48) or Omicron BA.1 (*n* = 49) breakthrough infection were examined, including 30 of 48 from Delta breakthrough and 30 of 49 from Omicron BA.1 breakthrough with IC_50_s lower than 1,000 ng/ml (neutralizing) against WT (Fig. S5 c). In general, there was no significant difference in the distribution of targeted epitopes among antibodies obtained following breakthrough infection as compared to those obtained after mRNA vaccination (Fig. S5 c).

All 288 WT RBD–binding antibodies were tested for neutralization in a SARS-CoV-2 pseudotype neutralization assay based on the WT SARS-CoV-2 spike (Robbiani et al., 2020; Schmidt et al., 2020). For comparison, we used a previously characterized set of antibodies isolated after the second (Cho et al., 2021) or the third vaccine dose (Muecksch et al., 2022). Potency against WT was considerably improved after Delta breakthrough infection compared to the second vaccine dose (Vax2; IC_50_ = 182 ng/ml vs. IC_50_ = 50 ng/ml, P = 0.0013; Fig. 3 b) but not compared to the third vaccine dose (IC_50_ = 98 ng/ml, P = 0.62; Fig. 3 b). In addition, there was no further improvement of neutralizing activity following Omicron BA.1 breakthrough infection compared to the third dose (IC_50_ = 73 ng/ml; P > 0.99; Fig. 3 b).

To examine whether and how neutralizing breadth evolves in vaccinees after Delta or Omicron BA.1 breakthrough infection, we analyzed the 288 newly expressed antibodies obtained from breakthrough individuals and 45 previously described antibodies obtained from Vax2 individuals (Cho et al., 2021) and measured their neutralizing activity against SARS-CoV-2 pseudoviruses carrying amino acid substitutions found in the Delta-RBD and Omicron BA.1 variant. In addition, 105 randomly selected antibodies from all four groups were tested against an Omicron BA.4/5 pseudovirus (Fig. 3 c). Neutralizing potency was generally lower against Omicron BA.1 compared to Delta pseudovirus. However, while antibodies obtained 5 mo after the second vaccine dose were not significantly more potent against Delta (IC_50_ = 181 ng/ml) vs. BA.1 pseudovirus (IC_50_ = 405 ng/ml; P = 0.20; Fig. 3 c), those obtained after subsequent Delta breakthrough infection neutralize Delta with 6.8-fold increased potency compared to BA.1 (P < 0.0001; Fig. 3 c). In contrast, the ratio of Delta vs. BA.1 IC_50_ in Vax3 antibodies was only 2.2 (P = 0.07; Fig. 3 c), while antibodies recovered after subsequent omicron breakthrough neutralized Delta and Omicron with similar potencies IC_50_ = 122 ng/ml for Delta vs. 148 ng/ml for Omicron (P = 0.92; Fig. 3 c).

Compared to the second vaccine dose, antibodies from Delta breakthrough infection showed increased potency against Delta pseudovirus (181 vs. 61 ng/ml, P = 0.047; Fig. 3 d). However, there was no significant improvement of antibody potency against Delta, Omicron BA.1, or Omicron BA.4/5 pseudovirus comparing 5 m-Vax2 versus Delta breakthrough antibodies. Moreover, there were only twofold differences that did not reach statistical significance when comparing Vax3 and Omicron breakthrough antibodies for Omicron BA.1 and BA.4/5 neutralization (Fig. 3 d). Notably, Omicron BA.4/5 showed the highest degree of neutralization resistance for all tested antibody groups (Fig. 3, c and d). Neutralizing activity of clonally related antibody pairs from participants C018 and C023 was measured against a panel of SARS-CoV-2 pseudoviruses harboring RBD amino acid substitutions representative of variants including Delta and Omicron BA.1. Most pairs of antibodies obtained from clones persisting between the third dose to the following Omicron BA.1 breakthrough infection showed little improvement in antibody breadth within the analyzed pairs (Table S4).

When comparing the fraction of antibodies showing neutralizing activity against Delta or Delta+WT, or Omicron BA.1 or Omicron BA.1+WT, or all three viruses (WT+Delta+Omicron BA.1), it became apparent that antibodies isolated after two vaccine doses and subsequent Delta breakthrough infection show the largest proportion of Delta-neutralizing antibodies. Conversely, antibodies isolated after the third vaccine dose and subsequent Omicron BA.1 breakthrough infection show the largest number of antibodies that neutralized all three pseudoviruses (Fig. 3 e and Fig. S5 d). Vax3 antibodies and Omicron BA.1 breakthrough antibodies were enriched for those neutralizing BA.4/5 with IC_50_ values of <1,000 ng/ml with 37 and 38% of all tested antibodies neutralizing BA.4/5, respectively, while only 17 and 27% of Vax2 and Delta breakthrough antibodies, respectively neutralized BA.4/5 (Fig. 3 f). Thus, in both cases tested, a third exposure to antigen increases memory antibody potency and breadth but a fourth exposure with Omicron BA.1 does little more when it occurs in the time frame measured in this study.

## Discussion

Omicron and its subvariants are reported to be more transmissible than any prior VoC and have spurred a resurgence of new cases worldwide (Mallapaty, 2022). While early reports suggested that Omicron may cause less severe illness, recent studies show variant-specific symptoms but similar virulence (Whitaker et al., 2022), and increased resistance to approved vaccine regimens (Nealon and Cowling, 2022).

We and others have shown that a third mRNA vaccine dose boosts plasma antibody responses to SARS-CoV-2 variants including Omicron BA.1 and increases the number, potency, and breadth of the antibodies found in the MBC compartment (Goel et al., 2022; Muecksch et al., 2022). Although the antibodies in plasma are generally not sufficient to prevent breakthrough infection, boosted individuals are protected against serious disease upon breakthrough infection (Kuhlmann et al., 2022; Nemet et al., 2022). Our findings suggest that a third exposure to antigen in the form of Delta breakthrough infection produces similar effects on the overall size of the memory compartment to a third mRNA vaccine dose, and specifically boosts strain-specific responses. In contrast, while a fourth antigen exposure by infection with Omicron elicits strain-specific memory, it has far more modest effects on the overall potency and breadth of MBC antibodies. The data suggest that a variant-specific mRNA vaccine boost will increase plasma neutralizing activity and memory B cells that are specific to the variant and closely related strains but may not elicit MBCs with better general potency or breadth than the Wuhan-Hu-1–based mRNA vaccine.

Antigenic variation between viral strains and the time interval between antigenic exposures are likely important contributors to the observed differences in immune responses. For example, the antigenic distance between Wuhan-Hu-1 and Delta is shorter than that between Wuhan-Hu-1 and Omicron BA.1 or between Delta (Liu et al., 2021) and Omicron BA.1 (Dejnirattisai et al., 2022), which could in part explain a more limited antibody response and less cross-reactive MBCs even after a fourth antigen exposure with Omicron BA.1. In addition, we found that Delta breakthrough infection resulted in similar Delta-specific antibody responses compared to a third mRNA vaccination or Omicron breakthrough infection. This may be partly due to shorter intervals between exposures in the Delta-BT cohort which is consistent with the notion that the duration after antigen exposure is associated with the continued evolution of the humoral response resulting in greater somatic hypermutation and breadth as well as increased potency (Cho et al., 2021; Gaebler et al., 2021; Sokal et al., 2021; Wang et al., 2021c).

The data highlight the challenges involved in selecting variant-specific vaccines in the absence of reliable information on the nature of the next emerging variant and suggest that a focus should be on designing vaccines with broader general activity against coronaviruses.

## Materials and methods

### Study participants

Participants were healthy adults that had been vaccinated with two or three doses of an mRNA vaccine (mRNA-1273 [Moderna] or BNT162b2 [Pfizer]) and reported breakthrough SARS-CoV-2 infection diagnosed by PCR or antigen testing. Breakthrough infection with Delta or Omicron variants were deduced based on the prevalent variant circulating in New York City at the time of infection (Gaebler et al., 2022). All participants provided written informed consent before participation in the study and the study was conducted in accordance with Good Clinical Practice. The study was performed in compliance with all relevant ethical regulations and the protocol (DRO-1006) for studies with human participants was approved by the Institutional Review Board of the Rockefeller University. For detailed participant characteristics, see Table S1.

### Blood samples processing and storage

Venous blood samples were collected into heparin and serum-gel monovette tubes by standard phlebotomy at The Rockefeller University. Peripheral blood mononuclear cells obtained from samples collected were further purified as previously reported by gradient centrifugation and stored in liquid nitrogen in the presence of FCS and DMSO (Gaebler et al., 2021; Robbiani et al., 2020). Heparinized serum and plasma samples were aliquoted and stored at −20°C or less. Prior to experiments, aliquots of plasma samples were heat-inactivated (56°C for 1 h) and then stored at 4°C.

### ELISAs

ELISAs (Amanat et al., 2020; Grifoni et al., 2020) were performed to evaluate antibodies binding to SARS-CoV-2 WT (Wuhan-Hu-1) RBD, and VoC Delta (B.1.617.2) RBD, and Omicron (BA.1) spike protein by coating of high-binding 96-half-well plates (3690; Corning) with 50 μl per well of a 1 μg/ml indicated protein solution in PBS overnight at 4°C. Plates were washed six times with washing buffer (1× PBS with 0.05% Tween-20; Sigma-Aldrich) and incubated with 170 μl per well blocking buffer (1× PBS with 2% BSA and 0.05% Tween-20; Sigma-Aldrich) for 1 h at room temperature. Immediately after blocking, plasma samples or mAbs were added in PBS and incubated for 1 h at room temperature. Plasma samples were assayed at a 1:66 starting dilution and 10 additional threefold serial dilutions.

10 μg/ml starting concentration was used to test mAbs followed by 10 additional fourfold serial dilutions. Plates were washed six times with washing buffer and then incubated with anti-human IgG secondary antibody conjugated to HRP (Jackson ImmunoResearch 109-036-088 109-035-129 and Sigma-Aldrich A0295) in blocking buffer at a 1:5,000 dilution. Plates were developed by addition of the HRP substrate, 3,3′,5,5′-tetramethylbenzidine (Thermo Fisher Scientific) for 10 min (plasma samples and mAbs). 50 μl of 1 M H_2_SO_4_ was used to stop the reaction and absorbance was measured at 450 nm with an ELISA microplate reader (FluoStar Omega, BMG Labtech) with Omega and Omega MARS software for analysis. A positive control (for anti-RBD ELISA, plasma from participant COV72, diluted 66.6-fold and 10 additional threefold serial dilutions in PBS; for anti-Omicron spike ELISA, plasma from B039 was used as a control) was added to every assay plate for normalization for plasma samples. The average of its signal was used for normalization of all the other values on the same plate with Excel software before calculating the area under the curve using Prism V9.1 (GraphPad). Negative controls of pre-pandemic plasma samples from healthy donors were used for validation (for more details, please see Robbiani et al., 2020). For mAbs, the ELISA EC_50_ was determined using four-parameter nonlinear regression (GraphPad Prism V9.1). EC_50_s above 1,000 ng/ml were considered non-binders.

### Proteins

The mammalian expression vector encoding the RBD of SARS-CoV-2 (GenBank MN985325.1; Spike [S] protein residues 319-539) was previously described (Barnes et al., 2020b).

### SARS-CoV-2–pseudotyped reporter virus

A panel of plasmids expressing RBD-mutant SARS-CoV-2 spike proteins in the context of pSARS-CoV-2-S_Δ19_ has been described (Cho et al., 2021; Muecksch et al., 2021; Wang et al., 2021c; Weisblum et al., 2020). Variant pseudoviruses resembling SARS-CoV-2 variants Delta (B.1.617.2) and Omicron BA.1 (B.1.1.529) have been described before (Cho et al., 2021; Schmidt et al., 2022; Wang et al., 2021b) and were generated by introduction of substitutions using synthetic gene fragments (IDT) or overlap extension PCR-mediated mutagenesis and Gibson assembly. Specifically, the variant-specific deletions and substitutions introduced were: Delta: T19R, Δ156-158, L452R, T478K, D614G, P681R, D950N; Omicron BA.1: A67V, Δ69-70, T95I, G142D, Δ143-145, Δ211, L212I, ins214EPE, G339D, S371L, S373P, S375F, K417N, N440K, G446S, S477N, T478K, E484A, Q493K, G496S, Q498R, N501Y, Y505H, T547K, D614G, H655Y, H679K, P681H, N764K, D796Y, N856K, Q954H, N969H, N969K, L981F; Omicron BA.2: T19I, L24S, del25-27, G142D, V213G, G339D, S371F, S373P, S375F, T376A, D405N, R408S, K417N, N440K, S477N, T478K, E484A, Q493R, Q498R, N501Y, Y505H, D614G, H655Y, N679K, P681H, N764K, D796Y, Q954H, N969K; Omicron BA.4/5: T19I, L24S, del25-27, del69-70, G142D, V213G, G339D, S371F, S373P, S375F, T376A, D405N, R408S, K417N, N440K, L452R, S477N, T478K, E484A, F486V, Q498R, N501Y, Y505H, D614G, H655Y, N679K, P681H, N764K, D796Y, Q954H, N969K.

Deletions/substitutions corresponding to VoC listed above were incorporated into a spike protein that also includes the R683G substitution, which disrupts the furin cleavage site and increases particle infectivity. Neutralizing activity against mutant pseudoviruses was compared to a WT SARS-CoV-2 spike sequence (NC_045512), carrying R683G where appropriate.

SARS-CoV-2–pseudotyped particles were generated as previously described (Robbiani et al., 2020; Schmidt et al., 2020). Briefly, 293T (CRL-11268) cells were obtained from ATCC, and the cells were transfected with pNL4-3ΔEnv-nanoluc and pSARS-CoV-2-S_Δ19_, particles were harvested 48 h after transfection, filtered, and stored at −80°C.

### Pseudotyped virus neutralization assay

Pre-pandemic negative control plasma from healthy donors, plasma from individuals who received mRNA vaccines and had Delta or Omicron BA.1 breakthrough infection, or mAbs were fivefold serially diluted and incubated with SARS-CoV-2–pseudotyped virus for 1 h at 37°C. The mixture was subsequently incubated with 293T_Ace2_ cells (Robbiani et al., 2020; for all WT neutralization assays) or HT1080/Ace2 cl14 cells (for all variant neutralization assays) for 48 h after which cells were washed with PBS and lysed with Luciferase Cell Culture Lysis 5× reagent (Promega). Nanoluc luciferase activity in lysates was measured using the Nano-Glo Luciferase Assay System (Promega) with the ClarioStar Microplate Multimode Reader (BMG). The relative luminescence units were normalized to those derived from cells infected with SARS-CoV-2–pseudotyped virus (Wang et al., 2021c) in the absence of plasma or mAbs. The NT_50_s for plasma or half-maximal and 90% inhibitory concentrations for mAbs (IC_50_ and IC_90_) were determined using four-parameter nonlinear regression (least squares regression method without weighting; constraints: top = 1, bottom = 0; GraphPad Prism).

### Biotinylation of viral protein for use in flow cytometry

Purified and Avi-tagged SARS-CoV-2 WT and Delta RBD was biotinylated using the Biotin-Protein Ligase-BIRA kit according to the manufacturer’s instructions (Avidity) as described before (Robbiani et al., 2020). Ovalbumin (Ova; A5503-1G; Sigma-Aldrich) was biotinylated using the EZ-Link Sulfo-NHS-LC-Biotinylation kit according to the manufacturer’s instructions (Thermo Fisher Scientific). Biotinylated Ova was conjugated to streptavidin-BV711 for single-cell sorts (563262; BD Biosciences) or to streptavidin-BB515 for phenotyping panel (564453; BD). WT RBD was conjugated to streptavidin-PE (554061; BD Biosciences) and streptavidin-AF647 (405237; Biolegend) for single-cell sorts, or streptavidin-BV421 (405225; Biolegend) and streptavidin-BV711 (563262; BD Biosciences) for phenotyping. Delta RBD was conjugated to streptavidin-PE (554061; BD Biosciences) and Omicron BA.1 RBD (SPD-C82E4; ACROBiosystems) was conjugated to streptavidin-AF647 (405237; Biolegend).

### Flow cytometry and single-cell sorting

Single-cell sorting by flow cytometry was described previously (Robbiani et al., 2020). Simply, peripheral blood mononuclear cells were enriched for B cells by negative selection using a pan-B-cell isolation kit according to the manufacturer’s instructions (130-101-638; Miltenyi Biotec). The enriched B cells were incubated in FACS buffer (1 × PBS, 2% FCS, 1 mM EDTA) with the following anti-human antibodies (all at 1:200 dilution): anti-CD20-PECy7 (335793; BD Biosciences), anti-CD3-APC-eFluro 780 (47-0037-41; Invitrogen), anti-CD8-APC-eFluor 780 (47-0086-42; Invitrogen), anti-CD16-APC-eFluor 780 (47-0168-41; Invitrogen), anti-CD14-APC-eFluor 780 (47-0149-42; Invitrogen), as well as Zombie NIR (423105; BioLegend), and fluorophore-labeled RBD and Ova for 30 min on ice. Single CD3^−^CD8^−^CD14^−^CD16^−^CD20^+^Ova^—^WT RBD-PE^+^-WT RBD-AF647^+^ B cells were sorted into individual wells of 96-well plates containing 4 μl of lysis buffer (0.5 × PBS, 10 mM dithiothreitol, 3,000 units/ml RNasin Ribonuclease Inhibitors; N2615; Promega) per well using a FACS Aria III and FACSDiva software (Becton Dickinson) for acquisition and FlowJo for analysis. The sorted cells were frozen on dry ice and then stored at −80°C or immediately used for subsequent RNA reverse transcription. For B cell phenotype analysis, in addition to above antibodies, B cells were also stained with following anti-human antibodies (all at 1:200 dilution): anti-IgD-BV650 (740594; BD), anti-CD27-BV786 (563327; BD Biosciences), anti-CD19-BV605 (302244; Biolegend), anti-CD71-PerCP-Cy5.5 (334114; Biolegend), anti-IgG-PECF594 (562538; BD), anti-IgM-AF700 (314538; Biolegend), and anti-IgA-Viogreen (130-113-481; Miltenyi Biotec).

### Antibody sequencing, cloning, and expression

Antibodies were identified and sequenced as described previously (Robbiani et al., 2020; Wang et al., 2021a). In brief, RNA from single cells was reverse-transcribed (SuperScript III Reverse Transcriptase; Invitrogen, 18080-044), and the cDNA was stored at −20°C or used for subsequent amplification of the variable *IGH*, *IGL*, and *IGK* genes by nested PCR and Sanger sequencing. Sequence analysis was performed using MacVector. Amplicons from the first PCR reaction were used as templates for sequence- and ligation-independent cloning into antibody expression vectors. Recombinant mAbs were produced and purified as previously described (Robbiani et al., 2020).

### BLI

BLI assays were performed as previously described (Robbiani et al., 2020). In brief, we used the Octet Red instrument (ForteBio) at 30°C with shaking at 1,000 r.p.m. Epitope-binding assays were performed with protein A biosensor (18-5010; ForteBio), following the manufacturer’s protocol “classical sandwich assay” as follows: (1) sensor check: sensors immersed 30 s in buffer alone (18-1,105; buffer ForteBio); (2) capture first antibody: sensors immersed 10 min with Ab1 at 10 μg/ml; (3) baseline: sensors immersed 30 s in buffer alone; (4) blocking: sensors immersed 5 min with IgG isotype control at 10 μg/ml; (5) baseline: sensors immersed 30 s in buffer alone; (6) antigen association: sensors immersed 5 min with RBD at 10 μg/ml; (7) baseline: sensors immersed 30 s in buffer alone; (8) association Ab2: sensors immersed 5 min with Ab2 at 10 μg/ml. Curve fitting was performed using the Fortebio Octet Data analysis software (ForteBio). Affinity measurement of anti–SARS-CoV-2 IgGs binding was corrected by subtracting the signal obtained from traces performed with IgGs in the absence of WT RBD. The kinetic analysis using protein A biosensor (as above) was performed as follows: (1) baseline: 60 s immersion in buffer; (2) loading: 200 s immersion in a solution with IgGs 10 μg/ml; (3) baseline: 200 s immersion in buffer; (4) association: 300 s immersion in solution with WT RBD at 20, 10, or 5 μg/ml; and (5) dissociation: 600 s immersion in buffer. Curve fitting was performed using a fast 1:1 binding model and the data analysis software (ForteBio). Mean *K*_D_ values were determined by averaging all binding curves that matched the theoretical fit with an R^2^ value ≥0.8.

### Computational analyses of antibody sequences

Antibody sequences were trimmed based on quality and annotated using Igblastn v.1.14 with IMGT domain delineation system. Annotation was performed systematically using Change-O toolkit v.0.4.540 (Gupta et al., 2015). Clonality of heavy and light chain was determined using DefineClones.py implemented by Change-O v.0.4.5 (Gupta et al., 2015). The script calculates the Hamming distance between each sequence in the data set and its nearest neighbor. Distances are subsequently normalized and to account for differences in junction sequence length, and clonality is determined based on a cut-off threshold of 0.15. Heavy and light chains derived from the same cell were subsequently paired, and clonotypes were assigned based on their V and J genes using in-house R and Perl scripts. All scripts and the data used to process antibody sequences are publicly available on GitHub (https://github.com/stratust/igpipeline/tree/igpipeline2_timepoint_v2).

The frequency distributions of human V genes in anti–SARS-CoV-2 antibodies from this study were compared to 131,284,220 IgH and IgL sequences generated by Soto et al. (2019) and downloaded from cAb-Rep (Guo et al., 2019), a database of human shared BCR clonotypes available at https://cab-rep.c2b2.columbia.edu/. We selected the IgH and IgL sequences from the database that are partially coded by the same V genes and counted them according to the constant region. The frequencies shown in Fig. S3 are relative to the source and isotype analyzed. We used the two-sided binomial test to check whether the number of sequences belonging to a specific *IGHV* or *IGLV* gene in the repertoire is different according to the frequency of the same IgV gene in the database. Adjusted P values were calculated using the false discovery rate correction. Significant differences are denoted with stars.

Nucleotide somatic hypermutation and complementarity-determining region (CDR3) length were determined using in-house R and Perl scripts. For somatic hypermutations, *IGHV* and *IGLV* nucleotide sequences were aligned against their closest germlines using Igblastn and the number of differences was considered to correspond to nucleotide mutations. The average number of mutations for V genes was calculated by dividing the sum of all nucleotide mutations across all participants by the number of sequences used for the analysis. GCTree (https://github.com/matsengrp/gctree; DeWitt et al., 2018) was further used to perform the phylogenetic trees construction. Each node represents a unique IgH and IgL combination and the size of each node is proportional to the number of identical sequences. The numbered nodes represent the unobserved ancestral genotypes between the germline sequence and the sequences on the downstream branch.

### Data presentation

Figures were arranged in Adobe Illustrator 2022.

### Online supplemental material

Fig. S1 shows plasma anti–Delta-RBD and anti–Omicron BA.1-Spike–binding activity after vaccination and following breakthrough infections with Delta or Omicron BA.1 variants. Fig. S2 shows flow cytometry gating strategy to phenotype or sort RBD-binding MBCs after vaccination and following breakthrough infections with Delta or Omicron BA.1 variants. Fig. S3 shows frequency of V gene usage of RBD-binding MBCs after vaccination and following breakthrough infections with Delta or Omicron BA.1 variants. Fig. S4 shows somatic hypermutations of antibody genes and phylogenetic trees of clonally related antibody families in this study. Fig. S5 shows additional characterization of antibodies’ affinity, epitopes, and neutralization breadth. Table S1 details individual characteristics for vaccinated participants who experienced Delta or Omicron BA.1 breakthrough infections. Table S2 details sequence information of all characterized RBD-binding MBCs from mRNA-vaccinated individuals who experienced breakthrough infections with Delta or Omicron BA.1. Table S3 provides information of a selected number of recombinant mAbs cloned from RBD-binding B cells. Table S4 provides binding and neutralization activities of recombinant mAbs that were clonally related.

## Supplementary Material

Table S1 details individual characteristics for vaccinated participants who experienced Delta or Omicron BA.1 breakthrough infections.

Table S2 details sequence information of all characterized RBD-binding MBCs from mRNA-vaccinated individuals who experienced breakthrough infections with Delta or Omicron BA.1.

Table S3 provides information of a selected number of recombinant mAbs cloned from RBD-binding B cells.

Table S4 provides binding and neutralization activities of recombinant mAbs that were clonally related.

## Data Availability

Data are provided in Tables S1, S2, S3, and S4. The raw sequencing data and computer scripts associated with Fig. 2 have been deposited at Github (https://github.com/stratust/igpipeline/tree/igpipeline2_timepoint_v2). This study also uses data from “A Public Database of Memory and Naive B-Cell Receptor Sequences” (https://doi.org/10.5061/dryad.35ks2), PDB (6VYB and 6NB6), cAb-Rep (https://cab-rep.c2b2.columbia.edu/), Sequence Read Archive (accession SRP010970), and from Soto et al. (2019). Computer code to process the antibody sequences is available at GitHub (https://github.com/stratust/igpipeline/tree/igpipeline2_timepoint_v2).

## References

[bib1] Amanat, F., D. Stadlbauer, S. Strohmeier, T.H.O. Nguyen, V. Chromikova, M. McMahon, K. Jiang, G.A. Arunkumar, D. Jurczyszak, J. Polanco, . 2020. A serological assay to detect SARS-CoV-2 seroconversion in humans. Nat. Med. 26:1033–1036. 10.1038/s41591-020-0913-5 32398876 PMC8183627

[bib2] Andrews, N., J. Stowe, F. Kirsebom, S. Toffa, T. Rickeard, E. Gallagher, C. Gower, M. Kall, N. Groves, A.M. O'Connell, . 2022. Covid-19 vaccine effectiveness against the omicron (B.1.1.529) variant. N. Engl. J. Med. 386:1532–1546. 10.1056/NEJMoa2119451 35249272 PMC8908811

[bib3] Barnes, C.O., C.A. Jette, M.E. Abernathy, K.A. Dam, S.R. Esswein, H.B. Gristick, A.G. Malyutin, N.G. Sharaf, K.E. Huey-Tubman, Y.E. Lee, . 2020a. SARS-CoV-2 neutralizing antibody structures inform therapeutic strategies. Nature. 588:682–687. 10.1038/s41586-020-2852-1 33045718 PMC8092461

[bib4] Barnes, C.O., A.P. West Jr, K.E. Huey-Tubman, M.A.G. Hoffmann, N.G. Sharaf, P.R. Hoffman, N. Koranda, H.B. Gristick, C. Gaebler, F. Muecksch, . 2020b. Structures of human antibodies bound to SARS-CoV-2 spike reveal common epitopes and recurrent features of antibodies. Cell. 182:828–842.e16. 10.1016/j.cell.2020.06.025 32645326 PMC7311918

[bib5] Cao, Y., A. Yisimayi, F. Jian, W. Song, T. Xiao, L. Wang, S. Du, J. Wang, Q. Li, X. Chen, . 2022. BA.2.12.1, BA.4 and BA.5 escape antibodies elicited by Omicron infection. Nature. 608:593–602. 10.1038/s41586-022-04980-y 35714668 PMC9385493

[bib6] Cele, S., L. Jackson, D.S. Khoury, K. Khan, T. Moyo-Gwete, H. Tegally, J.E. San, D. Cromer, C. Scheepers, D.G. Amoako, . 2022. Omicron extensively but incompletely escapes Pfizer BNT162b2 neutralization. Nature. 602:654–656. 10.1038/s41586-021-04387-1 35016196 PMC8866126

[bib7] Cho, A., F. Muecksch, D. Schaefer-Babajew, Z. Wang, S. Finkin, C. Gaebler, V. Ramos, M. Cipolla, P. Mendoza, M. Agudelo, . 2021. Anti-SARS-CoV-2 receptor-binding domain antibody evolution after mRNA vaccination. Nature. 600:517–522. 10.1038/s41586-021-04060-7 34619745 PMC8674133

[bib8] Dejnirattisai, W., J. Huo, D. Zhou, J. Zahradnik, P. Supasa, C. Liu, H.M.E. Duyvesteyn, H.M. Ginn, A.J. Mentzer, A. Tuekprakhon, . 2022. SARS-CoV-2 Omicron-B.1.1.529 leads to widespread escape from neutralizing antibody responses. Cell. 185:467–484.e15. 10.1016/j.cell.2021.12.046 35081335 PMC8723827

[bib9] DeWitt, W.S., 3rd, L. Mesin, G.D. Victora, V.N. Minin, and F.A. Matsen 4th. 2018. Using genotype Abundance to improve phylogenetic inference. Mol. Biol. Evol. 35:1253–1265. 10.1093/molbev/msy020 29474671 PMC5913685

[bib10] Gaebler, C., J. DaSilva, E. Bednarski, F. Muecksch, F. Schmidt, Y. Weisblum, K.G. Millard, M. Turroja, A. Cho, Z. Wang, . 2022. SARS-CoV-2 neutralization after mRNA vaccination and variant breakthrough infection. Open Forum Infect. Dis. 9:ofac227. 10.1093/ofid/ofac227 35818364 PMC9129198

[bib11] Gaebler, C., Z. Wang, J.C.C. Lorenzi, F. Muecksch, S. Finkin, M. Tokuyama, A. Cho, M. Jankovic, D. Schaefer-Babajew, T.Y. Oliveira, . 2021. Evolution of antibody immunity to SARS-CoV-2. Nature. 591:639–644. 10.1038/s41586-021-03207-w 33461210 PMC8221082

[bib12] Goel, R.R., M.M. Painter, K.A. Lundgreen, S.A. Apostolidis, A.E. Baxter, J.R. Giles, D. Mathew, A. Pattekar, A. Reynaldi, D.S. Khoury, . 2022. Efficient recall of Omicron-reactive B cell memory after a third dose of SARS-CoV-2 mRNA vaccine. Cell. 185:1875–1887.e8. 10.1016/j.cell.2022.04.009 35523182 PMC8989683

[bib13] Grifoni, A., D. Weiskopf, S.I. Ramirez, J. Mateus, J.M. Dan, C.R. Moderbacher, S.A. Rawlings, A. Sutherland, L. Premkumar, R.S. Jadi, . 2020. Targets of T cell responses to SARS-CoV-2 coronavirus in humans with COVID-19 disease and unexposed individuals. Cell. 181:1489–1501.e15. 10.1016/j.cell.2020.05.015 32473127 PMC7237901

[bib14] Guo, Y., K. Chen, P.D. Kwong, L. Shapiro, and Z. Sheng. 2019. cAb-Rep: a database of curated antibody repertoires for exploring antibody diversity and predicting antibody prevalence. Front. Immunol. 10:2365 10.3389/fimmu.2019.02365 31649674 PMC6794461

[bib15] Gupta, N.T., J.A. Vander Heiden, M. Uduman, D. Gadala-Maria, G. Yaari, and S.H. Kleinstein. 2015. Change-O: A toolkit for analyzing large-scale B cell immunoglobulin repertoire sequencing data. Bioinformatics. 31:3356–3358. 10.1093/bioinformatics/btv359 26069265 PMC4793929

[bib16] Hachmann, N.P., J. Miller, A.-r.Y. Collier, J.D. Ventura, J. Yu, M. Rowe, E.A. Bondzie, O. Powers, N. Surve, K. Hall, and D.H. Barouch. 2022. Neutralization Escape by the SARS-CoV-2 Omicron Variants BA.2.12.1 and BA.4/BA.5. medRxiv. (Preprint Posted May 19, 2022). 10.1101/2022.05.16.22275151 PMC9258748 35731894

[bib17] Kaku, C.I., A.J. Bergeron, C. Ahlm, J. Normark, M. Sakharkar, M.N.E. Forsell, and L.M. Walker. 2022. Recall of pre-existing cross-reactive B cell memory following Omicron BA.1 breakthrough infection. Sci. Immunol. 7:eabq3511 35549299 10.1126/sciimmunol.abq3511 PMC9097882

[bib18] Kuhlmann, C., C.K. Mayer, M. Claassen, T. Maponga, W.A. Burgers, R. Keeton, C. Riou, A.D. Sutherland, T. Suliman, M.L. Shaw, and W. Preiser. 2022. Breakthrough infections with SARS-CoV-2 omicron despite mRNA vaccine booster dose. Lancet. 399:625–626. 10.1016/S0140-6736(22)00090-3 35063123 PMC8765759

[bib19] Liu, C., H.M. Ginn, W. Dejnirattisai, P. Supasa, B. Wang, A. Tuekprakhon, R. Nutalai, D. Zhou, A.J. Mentzer, Y. Zhao, . 2021. Reduced neutralization of SARS-CoV-2 B.1.617 by vaccine and convalescent serum. Cell. 184:4220–4236.e13. 10.1016/j.cell.2021.06.020 34242578 PMC8218332

[bib20] Madhi, S.A., G. Kwatra, J.E. Myers, W. Jassat, N. Dhar, C.K. Mukendi, A.J. Nana, L. Blumberg, R. Welch, N. Ngorima-Mabhena, and P.C. Mutevedzi. 2022. Population immunity and covid-19 severity with omicron variant in South Africa. N. Engl. J. Med. 386:1314–1326. 10.1056/NEJMoa2119658 35196424 PMC8908853

[bib21] Mallapaty, S. 2022. COVID-19: How Omicron overtook Delta in three charts. Nature. 10.1038/d41586-022-00632-3 35246640

[bib22] Muecksch, F., Z. Wang, A. Cho, C. Gaebler, T. Ben Tanfous, J. DaSilva, E. Bednarski, V. Ramos, S. Zong, B. Johnson, . 2022. Increased memory B cell potency and breadth after a SARS-CoV-2 mRNA boost. Nature. 607:128–134. 10.1038/s41586-022-04778-y 35447027 PMC9259484

[bib23] Muecksch, F., Y. Weisblum, C.O. Barnes, F. Schmidt, D. Schaefer-Babajew, Z. Wang, J.C. Lorenzi, A.I. Flyak, A.T. DeLaitsch, K.E. Huey-Tubman, . 2021. Affinity maturation of SARS-CoV-2 neutralizing antibodies confers potency, breadth, and resilience to viral escape mutations. Immunity. 54:1853–1868.e7. 10.1016/j.immuni.2021.07.008 34331873 PMC8323339

[bib24] Nealon, J., and B.J. Cowling. 2022. Omicron severity: Milder but not mild. Lancet. 399:412–413. 10.1016/S0140-6736(22)00056-3 35065007 PMC8769661

[bib25] Nemet, I., L. Kliker, Y. Lustig, N. Zuckerman, O. Erster, C. Cohen, Y. Kreiss, S. Alroy-Preis, G. Regev-Yochay, E. Mendelson, and M. Mandelboim. 2022. Third BNT162b2 vaccination neutralization of SARS-CoV-2 omicron infection. N. Engl. J. Med. 386:492–494. 10.1056/NEJMc2119358 34965337 PMC8823651

[bib26] Nutalai, R., D. Zhou, A. Tuekprakhon, H.M. Ginn, P. Supasa, C. Liu, J. Huo, A.J. Mentzer, H.M.E. Duyvesteyn, A. Dijokaite-Guraliuc, . 2022. Potent cross-reactive antibodies following Omicron breakthrough in vaccinees. Cell. 185:2116–2131.e18. 10.1016/j.cell.2022.05.014 35662412 PMC9120130

[bib27] Park, Y.-J., D. Pinto, A.C. Walls, Z. Liu, A.D. Marco, F. Benigni, F. Zatta, C. Silacci-Fregni, J. Bassi, K.R. Sprouse, . 2022. Imprinted antibody responses against SARS-CoV-2 Omicron sublineages. bioRxiv. (Preprint Posted May 10, 2022). 10.1101/2022.05.08.491108 PMC12945441 36264829

[bib28] Quandt, J., A. Muik, N. Salisch, B.G. Lui, S. Lutz, K. Kruger, A.K. Wallisch, P. Adams-Quack, M. Bacher, A. Finlayson, . 2022. Omicron BA.1 breakthrough infection drives cross-variant neutralization and memory B cell formation against conserved epitopes. Sci. Immunol. eabq2427. 10.1126/sciimmunol.abq2427 35653438 PMC9162083

[bib29] Richardson, S.I., V.S. Madzorera, H. Spencer, N.P. Manamela, M.A. van der Mescht, B.E. Lambson, B. Oosthuysen, F. Ayres, Z. Makhado, T. Moyo-Gwete, . 2022. SARS-CoV-2 Omicron triggers cross-reactive neutralization and Fc effector functions in previously vaccinated, but not unvaccinated, individuals. Cell Host Microbe. 30:880–886.e4. 10.1016/j.chom.2022.03.029 35436444 PMC8947963

[bib30] Robbiani, D.F., C. Gaebler, F. Muecksch, J.C.C. Lorenzi, Z. Wang, A. Cho, M. Agudelo, C.O. Barnes, A. Gazumyan, S. Finkin, . 2020. Convergent antibody responses to SARS-CoV-2 in convalescent individuals. Nature. 584:437–442. 10.1038/s41586-020-2456-9 32555388 PMC7442695

[bib31] Schmidt, F., F. Muecksch, Y. Weisblum, J. Da Silva, E. Bednarski, A. Cho, Z. Wang, C. Gaebler, M. Caskey, M.C. Nussenzweig, . 2022. Plasma neutralization of the SARS-CoV-2 Omicron variant. N. Engl. J. Med. 386:599–601. 10.1056/NEJMc2119641 35030645 PMC8757565

[bib32] Schmidt, F., Y. Weisblum, F. Muecksch, H.-H. Hoffmann, E. Michailidis, J.C.C. Lorenzi, P. Mendoza, M. Rutkowska, E. Bednarski, C. Gaebler, . 2020. Measuring SARS-CoV-2 neutralizing antibody activity using pseudotyped and chimeric viruses. J. Exp. Med. 217:e20201181. 10.1084/jem.20201181 32692348 PMC7372514

[bib33] Seaman, M.S., M.J. Siedner, J. Boucau, C.L. Lavine, F. Ghantous, M.Y. Liew, J. Mathews, A. Singh, C. Marino, J. Regan, . 2022. Vaccine Breakthrough Infection with the SARS-CoV-2 Delta or Omicron (BA.1) Variant Leads to Distinct Profiles of Neutralizing Antibody Responses. medRxiv. (Preprint Posted March 03, 2022). 10.1101/2022.03.02.22271731 PMC9675445 36214224

[bib34] Servellita, V., A.M. Syed, M.K. Morris, N. Brazer, P. Saldhi, M. Garcia-Knight, B. Sreekumar, M.M. Khalid, A. Ciling, P.Y. Chen, . 2022. Neutralizing immunity in vaccine breakthrough infections from the SARS-CoV-2 Omicron and Delta variants. Cell. 185:1539–1548.e5. 10.1016/j.cell.2022.03.019 35429436 PMC8930394

[bib35] Sokal, A., P. Chappert, G. Barba-Spaeth, A. Roeser, S. Fourati, I. Azzaoui, A. Vandenberghe, I. Fernandez, A. Meola, M. Bouvier-Alias, . 2021. Maturation and persistence of the anti-SARS-CoV-2 memory B cell response. Cell. 184:1201–1213.e14. 10.1016/j.cell.2021.01.050 33571429 PMC7994111

[bib36] Soto, C., R.G. Bombardi, A. Branchizio, N. Kose, P. Matta, A.M. Sevy, R.S. Sinkovits, P. Gilchuk, J.A. Finn, and J.E. Crowe. 2019. High frequency of shared clonotypes in human B cell receptor repertoires. Nature. 566:398–402. 10.1038/s41586-019-0934-8 30760926 PMC6949180

[bib37] Supasa, P., D. Zhou, W. Dejnirattisai, C. Liu, A.J. Mentzer, H.M. Ginn, Y. Zhao, H.M.E. Duyvesteyn, R. Nutalai, A. Tuekprakhon, . 2021. Reduced neutralization of SARS-CoV-2 B.1.1.7 variant by convalescent and vaccine sera. Cell. 184:2201–2211.e7. 10.1016/j.cell.2021.02.033 33743891 PMC7891044

[bib38] Victora, G.D., and M.C. Nussenzweig. 2022. Germinal centers. Annu. Rev. Immunol. 40:413–442. 10.1146/annurev-immunol-120419-022408 35113731

[bib39] Wang, Z., J.C.C. Lorenzi, F. Muecksch, S. Finkin, C. Viant, C. Gaebler, M. Cipolla, H.-H. Hoffmann, T.Y. Oliveira, D.A. Oren, . 2021a. Enhanced SARS-CoV-2 neutralization by dimeric IgA. Sci. Trans. Med. 13:eabf1555. 10.1126/scitranslmed.abf155 PMC7857415 33288661

[bib40] Wang, Z., F. Muecksch, D. Schaefer-Babajew, S. Finkin, C. Viant, C. Gaebler, H.-H. Hoffmann, C.O. Barnes, M. Cipolla, V. Ramos, . 2021b. Naturally enhanced neutralizing breadth against SARS-CoV-2 one year after infection. Nature. 595:426–431. 10.1038/s41586-021-03696-9 34126625 PMC8277577

[bib41] Wang, Z., F. Schmidt, Y. Weisblum, F. Muecksch, C.O. Barnes, S. Finkin, D. Schaefer-Babajew, M. Cipolla, C. Gaebler, J.A. Lieberman, . 2021c. mRNA vaccine-elicited antibodies to SARS-CoV-2 and circulating variants. Nature. 592:616–622. 10.1038/s41586-021-03324-6 33567448 PMC8503938

[bib42] Weisblum, Y., F. Schmidt, F. Zhang, J. DaSilva, D. Poston, J.C. Lorenzi, F. Muecksch, M. Rutkowska, H.-H. Hoffmann, E. Michailidis, . 2020. Escape from neutralizing antibodies by SARS-CoV-2 spike protein variants. Elife. 9:e61312. 10.7554/eLife.61312 33112236 PMC7723407

[bib43] Whitaker, M., J. Elliott, B. Bodinier, W. Barclay, H. Ward, G. Cooke, C.A. Donnelly, M. Chadeau-Hyam, and P. Elliott. 2022. Variant-specific symptoms of COVID-19 among 1,542,510 People in England. medRxiv. (Preprint Posted May 23, 2022). 10.1101/2022.05.21.22275368 PMC9651890 36369151

[bib44] Wolter, N., W. Jassat, S. Walaza, R. Welch, H. Moultrie, M. Groome, D.G. Amoako, J. Everatt, J.N. Bhiman, C. Scheepers, . 2022. Early assessment of the clinical severity of the SARS-CoV-2 omicron variant in South Africa: A data linkage study. Lancet. 399:437–446. 10.1016/S0140-6736(22)00017-4 35065011 PMC8769664

[bib45] World Health Organization. 2022. WHO SAGE roadmap for Prioritizing Uses of COVID-19 Vaccines: an approach to Optimize the Global impact of COVID-19 Vaccines, Based on Public Health Goals, Global and National Equity, and Vaccine access and Coverage scenarios. World Health Organization, Geneva. first issued 20 October 2020, updated: 13 November 2020, updated: 16 July 2021, latest update: 21 January 2022

[bib46] Yuan, M., H. Liu, N.C. Wu, C.-C.D. Lee, X. Zhu, F. Zhao, D. Huang, W. Yu, Y. Hua, H. Tien, . 2020. Structural basis of a shared antibody response to SARS-CoV-2. Science. 369:1119–1123. 10.1126/science.abd2321 32661058 PMC7402627

